# Co-Occurrence of *Borrelia burgdorferi* Sensu Lato and *Babesia* spp. DNA in *Ixodes ricinus* Ticks Collected from Vegetation and Pets in the City of Poznań, Poland

**DOI:** 10.3390/pathogens13040307

**Published:** 2024-04-10

**Authors:** Justyna Liberska, Jerzy Franciszek Michalik, Julia Olechnowicz, Miroslawa Dabert

**Affiliations:** 1 Molecular Biology Techniques Laboratory, Faculty of Biology, Adam Mickiewicz University, 61-614 Poznan, Poland; julia.olechnowicz@amu.edu.pl (J.O.); mirkad@amu.edu.pl (M.D.); 2 Department of Animal Morphology, Faculty of Biology, Adam Mickiewicz University, 61-614 Poznan, Poland; jerzy.michalik@amu.edu.pl

**Keywords:** *Ixodes ricinus*, *Borrelia burgdorferi* s.l., *Babesia microti*, *Babesia venatorum*, *Babesia canis*, *Borrelia miyamotoi*, co-infections, babesiosis, urban green areas

## Abstract

Here, we described the prevalence of *Borrelia burgdorferi* s.l. and *Babesia* species found in mono- and double infections among *Ixodes ricinus* ticks occurring in urban areas of the city of Poznań, Poland. We tested 1029 host-seeking ticks and 1268 engorged ticks removed from pet animals. *Borrelia afzelii* and *B. garinii* prevailed both in ticks from vegetation (3.7% and 3.7%, respectively) and from pets (3.7% and 0.6%, respectively). *Babesia canis* and *Ba. microti* were the most prevalent in host-seeking (2.6% and 1.4%, respectively) and feeding ticks (2.8% and 2.2%, respectively). *Babesia microti* sequences proved to be identical to the human pathogenic *Ba. microti* genotype “Jena/Germany”. Sequences of the rarest piroplasm *Ba. venatorum* (0.7%) were identical with those isolated from European patients. About 1.0% of tested ticks yielded dual infections; in host-seeking ticks, *Ba. canis* prevailed in co-infections with *B. afzelii* and *B. garinii*, whereas *Ba. microti* and *B. afzelii* dominated in double-infected feeding ticks. Dual infections, even with a low prevalence, pose a challenge for differential diagnosis in patients with acute febrile disease after a tick bite. The finding of *Ba. canis* in both tick groups suggests that *I. ricinus* could be involved in the circulation of this piroplasm.

## 1. Introduction

Green spaces inside European towns, such as parks, leisure time areas for hiking and biking, botanic gardens, private properties with gardens, cemeteries, and urban forests, create favorable environmental (e.g., temperature and humidity) conditions for *Ixodes ricinus*, the most widespread and important vector of tick-borne pathogens (TBPs) in Central Europe. One of the key factors influencing the survival and maintenance of local tick populations is their access to appropriate and abundant tick bloodmeal hosts [1]. Within urban green areas, immature stages of this primarily forest-dwelling tick species feed mostly on small rodents, ground-feeding passerines, and hedgehogs, which are additionally important hosts of adult female ticks. The latter also feed on urban pet populations represented by dogs and cats, including stray animals. The observed ongoing increase in the number of pets in towns suggests that this group of mammals appears to be increasingly important for the persistence and size of tick populations in these ecologically altered habitats. Some of the vertebrate species acting as maintenance hosts for *I. ricinus* may concurrently serve as reservoir hosts of TBPs. Their high or low abundance and species composition in urban habitats influence the level of infection of local tick populations and are critical for public health importance [1]. Therefore, investigations regarding the prevalence of TBP-infected ticks are necessary to establish or predict the emergence of active endemic foci of tick-borne diseases. It is particularly crucial because there is still a lack of comprehensive knowledge on the eco-epidemiology of these infections in urban ecosystems and our understanding of how urbanization affects pathogen–host–vector relationships [2]. 

Ticks can carry two or more pathogenic microorganisms with a subsequent high likelihood of co-transmission to humans or animals [3,4]. *Ixodes ricinus* ticks infected with various bacteria, i.e., spirochetes of the *Borrelia burgdorferi* sensu lato (s.l.) complex, the agent of Lyme borreliosis (LB) or *Anaplasma phagocytophilum*, the agent of human anaplasmosis, are regularly found in urban and suburban areas across Europe [5,6,7]. Furthermore, *B. burgdorferi* s.l. spirochetes and intraerythrocytic parasites of the protozoan genus *Babesia* (Apicomplexa: Piroplasmida), including *Ba. microti*, *Ba. divergens*, *Ba. venatorum*, and *Ba. duncani* (present only in North America), can co-occur and be co-transmitted by ticks of the *Ixodes ricinus* species complex [8]. These protozoan pathogens are responsible for human babesiosis, causing a febrile hemolytic anemia that is generally asymptomatic or self-limiting in healthy humans; however, it is a serious health concern in splenectomized, immunocompromised patients [9]. In North America, the major agent of human babesiosis is *Ba. microti*, a parasite associated with small mammals serving as primary reservoir hosts. There is eco-epidemiological evidence indicating that co-infections *B. burgdorferi* s.l. and *Ba. microti* among ticks and *Peromyscus leucopus* mice may contribute to the emergence and expansion of *Ba. microti* in the enzootic cycle. Ecological models demonstrated the strongest effects when the prevalence of *B. burgdorferi* in mice was high [10]. Recent research clearly demonstrated that infection of *I. scapularis* ticks with *B. burgdorferi* s.l. spirochetes increases the likelihood of infection with *Ba. microti* as well as *A. phagocytophilum* compared with borreliae-free ticks [11]. Furthermore, according to Zembsch et al. [12], host-seeking *I. scapularis* ticks that are infected with *Ba. microti* are more likely to be co-infected with *B. burgdorferi* than expected if the pathogens were transmitted independently. This implies that such positive pathogen–vector–host interactions of both tick-borne agents may favor their emergence and maintenance in local tick populations [13]. Co-infections may also change clinical symptoms, course, and severity of tick-associated disease in humans and animals compared to those induced by a single infection [14,15,16]. Patients co-infected with *B. burgdorferi* s.l. and *Ba. microti* suffer from significantly more diverse, intense, and persisting disease symptoms compared to those infected with each pathogen separately [17,18]. It has been shown that *Ba. microti* weakens adaptive immunity and increases the severity of LB [19]. Furthermore, human co-infection with *Ba. microti* and *B. burgdorferi* s.l. seems to be a serious clinical problem because of the difficulties in diagnosis and treatment, since the antibiotics used to treat borreliae are ineffective against *Ba. microti* [20]. In Europe, most cases of human babesiosis are attributed to *Ba. divergens*, usually a cattle parasite or less frequently to *Ba. venatorum*, for which the roe deer is the main reservoir host. Interestingly, European genotypes of *Ba. microti* infecting humans are not as infectious or pathogenic as those in the USA [21].

The aim of our study was to ascertain the co-occurrence of *B. burgdorferi* s.l. spirochetes and *Babesia* piroplasms in *I. ricinus* ticks collected from vegetation as well as from dogs and cats in urban areas of the city of Poznań, west-central Poland.

## 2. Materials and Methods

### 2.1. Tick Collection and Identification and DNA Extraction

Host-seeking ticks were collected by sweeping up the vegetation up to 1 m with a 1 m^2^ flannel flag along paths in five forested green areas used for recreational activities and as walking areas for dogs in the city of Poznań. Ticks were collected from May to September 2017 and in April 2018. The five study sites included Morasko Adam Mickiewicz University Campus, Sołacki and Citadel city parks, and two urban forests localized around Rusałka and Malta lakes; for details, see [22].

Ticks feeding on dogs and cats were collected during a three-year survey (April to October 2015, March to November 2016, and March to September 2017) in 17 veterinary clinics in Poznań (for details, see [22]). In this study, we tested ticks derived from animals which did not travel outside the city, based on information from questionnaires.

All ticks were preserved in 96% ethanol until DNA extraction. Adult ticks were identified to the species level using morphological characteristics [23]. *Ixodes* nymphs were identified using DNA barcoding based on cytochrome *c* oxidase subunit I (COI) amplification and next-generation sequencing (NGS) of the amplicons [22,24]. Ticks were tested individually. DNA extraction from host-seeking ticks was performed with the ammonium hydroxide method, whereas DNA from pet-derived ticks was isolated using a silica-column method. Details concerning DNA extractions are described by Liberska et al. [22].

### 2.2. Screening for Borrelia burgdorferi s.l. DNA

Host-seeking ticks were initially screened for *B. burgdorferi* s.l. DNA by amplification and sequencing of the V4 hypervariable region of the 16S rRNA gene (V4 16S). Details concerning PCR primers, library construction, next-generation sequencing (NGS), and sequence data analysis were described previously [25]. Host-seeking and feeding ticks positive for *B. burgdorferi* s.l. V4 16S were retested by amplification and sequencing the *flaB* gene fragment using two primer sets 132f/905r and 220f/823r [26]. Protocols for PCR reactions and Sanger sequencing followed [25].

### 2.3. Screening for Babesia spp. DNA

*Babesia* spp. DNA was detected by amplification and sequencing of the 18S rRNA gene fragment using nested PCR and Sanger sequencing or by NGS of the same target DNA. For the nested PCR, we used the RIB-19/RIB-20 primer set for the first-round reaction [27], and the P3/BabR3 primer set for the second-round reaction [22,28]. Primer sequences and protocols for nested PCR and Sanger sequencing are described by Liberska et al. [22].

The second approach for the detection of *Babesia* DNA was conducted with the use of the P3/BabR3 primer set fused with dual-indexed Ion Torrent adapters to generate *Babesia*-specific amplicons for NGS sequencing. Details concerning library construction, Ion Torrent sequencing, and sequence data analysis were described previously [25].

### 2.4. Statistical Analysis

Data analysis was performed using STATISTICA software version 6.0 (StatSoft Inc., Tulsa, OK, USA). Rates of infection were analyzed using Chi-squared test χ^2^ and Fisher exact test. A *p* < 0.05 was considered statistically significant.

### 2.5. Sequence Analysis

Sanger sequence chromatograms were checked for accuracy in GeneiousR11.1.5 (Biomatters Ltd., Auckland, New Zealand) and contigs were assembled manually in GeneDoc sequence editing tool version 2.7 [29]. Amplicon sequence variants (ASVs) and operational taxonomic units (OTUs) obtained from NGS data were compared to those available in the GenBank using BLASTN (https://blast.ncbi.nlm.nih.gov, accessed on 28 February 2024) and the megablast algorithm. We used 100% identity threshold to determine *Borrelia* and *Babesia* species because they were determined from rDNA sequences. The identity threshold for tick species determination was lowered to 97% due to intra-species variability of the COI marker.

### 2.6. Phylogenetic Analysis

For phylogenetic analysis, we used 49 V4 16S sequences (Table S1), including ASVs found in this study (*n* = 18), V4 16S sequences identical to the ASVs found in this study and sourced from reference *B. burgdorferi* s.l. genomes deposited in GenBank (*n* = 18), and representatives of *Borrelia* RF group (*n* = 13) used to root the tree. Sequences were aligned manually in GeneDoc. The final alignment of 253 nucleotide positions was used to construct phylogenetic trees, which were built using FastTree 2.1.11 and MrBayes 3.2.6, both using the GTR+G model, as implemented in Geneious Prime 2023.2.1 (Biomatters Ltd.). Statistical supports for branches were estimated by Shimodaira–Hasegawa test (SH) [30] and Bayesian posterior probability (PP). Trees were edited in Mega7 [31] and Corel Draw v. X5.

## 3. Results

### 3.1. Collection and Identification of Ticks

A total of 2297 I. ricinus ticks, including 1029 host-seeking individuals (460 nymphs, 289 females, and 280 males) and 1268 feeding female ticks removed from 1115 tick-infested companion animals, were collected during this study (Table 1 and Table 2). In the case of pet-derived ticks sampled in veterinary clinics, 711 were removed from 609 dogs, 153 from 117 cats, and 404 from 389 undefined animals, i.e., without giving information about host species (Table 1).

### 3.2. Detection of Borrelia burgdorferi s.l. DNA

Based on V4 16S and *flaB* gene fragments, DNA of *B. burgdorferi* s.l. was detected in 90 (8.7%) of the 1059 host-seeking *I. ricinus* ticks. The bacterium was found in nymphs, males, and females with prevalences of 6.3%, 9.6%, and 11.8%, respectively (Table 2). Four out of the five collection sites yielded ticks that tested positive for *Borrelia* spirochetes (range between 3.0% and 40.6%). Sequencing of the amplified *flaB* gene fragments revealed four *Borrelia* species: *B. afzelii*, *B. garinii*, *B. lusitaniae*, and *B. valaisiana* (Table 2). The first two prevailed and reached the same prevalence of 3.7%, followed by *B. lusitaniae* (0.9%), *B. valaisiana* (0.3%), and *Borrelia* undetermined species (0.2%).

**Table 2:** The prevalence of *Borrelia burgdorferi* s.l. and *Babesia* spp. in single and double infections found in 1029 host-seeking *I. ricinus* ticks collected in urban areas of the city of Poznań.

	Females	Males	Nymphs	Total (%)
***Borrelia* spp.**				
*B. afzelii*	10	17	11	38 (3.7)
*B. garinii*	19	8	11	38 (3.7)
*B. lusitaniae*	2	0	7	9 (0.9)
*B.* *valaisiana*	2	1	0	3 (0.3)
*Borrelia* spp.	1	1	0	2 (0.2)
Total	34/289 (11.8)	27/280 (9.6)	29/460 (6.3)	90/1029 (8.7)
***Babesia* spp.**				
*Babesia microti*	10	9	8	27 (2.6)
*Babesia canis*	8	3	3	14 (1.4)
*Babesia venatorum*	3	1	0	4 (0.4)
Total	21/289 (7.3)	13/280 (4.6)	11/460 (2.4)	45/1029 (4.4)
***co-infections***				
*B. afzelii + Ba. canis*	1	3	0	4 (0.4)
*B. garinii + Ba. canis*	1	3	0	4 (0.4)
*B. lusitaniae + Ba. venatorum*	2	0	0	2 (0.2)
*B. lusitaniae + Ba. microti*	1	0	0	1 (0.1)
Total	5	6	0	11/1029 (1.1)

Among the 1268 *I. ricinus* females collected from animals, DNA *B. burgdorferi* s.l. was detected in 4.7% (*n* = 59) individuals, and this prevalence was almost two-fold lower in comparison to host-seeking ticks (8.7%, χ^2^ test, *p* = 0.001). Infected ticks were found in all animal groups: dogs (4.8%), cats (3.3%), and undefined pets (5.0%). Five spirochete species were identified with a clear predominance of *B. afzelii* (3.7%; *n* = 47) followed by *B. garinii* (0.6%; *n* = 7), *B. spielmanii* (0.2%; *n* = 3), *B. lusitaniae* (0.1%; *n* = 1), and *B. valaisiana* (0.1%). Except for *B. valaisiana*, the remaining four species were detected in dog-derived ticks. The five infected ticks collected from cats harbored only *B. afzelii*, whereas 20 individuals from the group of undefined pets were infected with *B. afzelii* (*n* = 16), *B. garinii* (*n* = 3), and *B. valaisiana.*

### 3.3. Genetic Diversity of B. burgdorferi s.l. V4 16S Amplicon Sequence Variants

**Figure 1:**
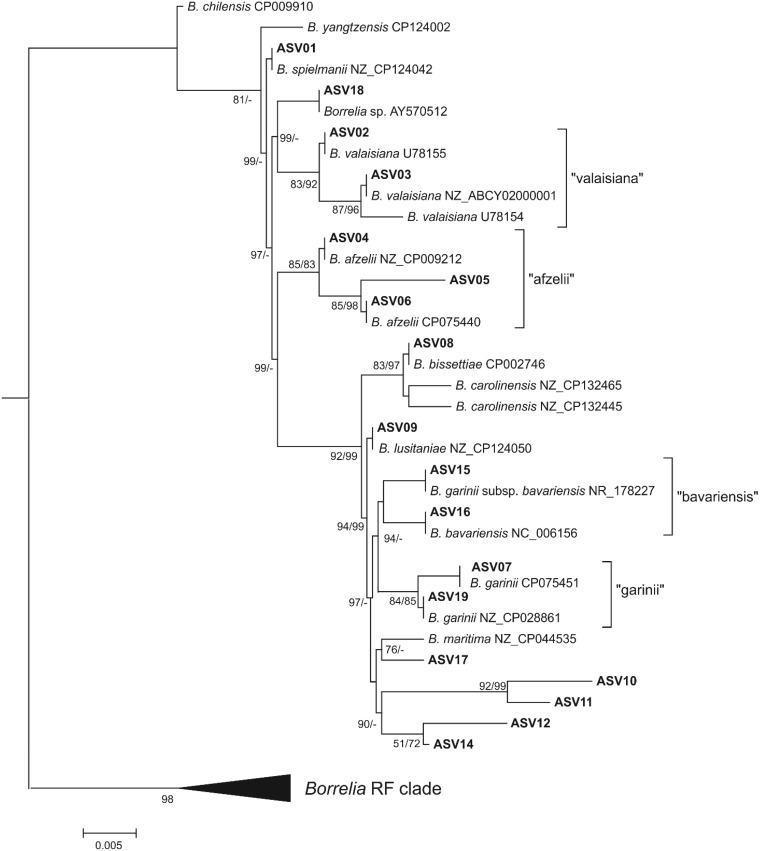
Phylogenetic analysis of amplicon sequence variants (ASVs) of the V4 region of the 16S rRNA gene found in this study. We show the tree reconstructed by the FastTree method because Bayesian tree (Figure S1) was largely polytomous; however, Bayesian inference (BI) analysis in many cases revealed the same relationships among ASVs and reference sequences. Numbers near branches show support values (SH/PP); only values > 50% are present; “-” not reconstructed in the BI analysis.

In total, 18 different *B. burgdorferi* s.l. V4 16S ASVs were found in questing ticks (GenBank acc. nos. PP406290-PP407999; Table S1). For most ASVs, identical sequences in species reference genomes published in GenBank could be assigned (Figure 1 and Figure S1). In Figure 1, we show the result of the FastTree analysis because the Bayesian tree (Figure S1) was largely polytomous. Both phylogenetic analyses enabled the assignment of one of the previously unknown variants, ASV05, to *B*. *afzelii* with a relatively high support (85% SH, 83 PP). Another new variant, ASV17, was reconstructed as a sister to the V4 16S sequence from the *B*. *maritima* reference genome, although only in the FastTree analysis and with moderate support (76% SH). The remaining four new variants (ASV10-12 and ASV14) were reconstructed by the FastTree analysis as a well-supported (90% SH) separate clade. These ASVs tended to co-occur with other *B. burgdorferi* s.l. V4 16S ASVs either in a relatively large number of ticks (ASV10 and ASV 14 in nine positive samples each) or in a predominant number of reads (ASV11). Amplicon sequence variant 12 was found in only one sample and in co-occurrence with ASV14; however, both sequences differed at two variable nucleotide positions, indicating that ASV12 cannot be considered an NGS artifact of the ASV14 amplicon.

Analysis of the *flaB* gene sequence was unable to unambiguously assign most of the V4 16S ASVs to the species. Samples positive for *B. burgdorferi* s.l. V4 16S DNA that clustered basally at our phylogenetic tree (ASV01-03 and ASV18) failed in PCR amplification of the *flaB* gene fragment. Moreover, most of the ASVs occurred in co-infections, making it impossible to assign a specific *flaB* gene sequence to a specific V4 16S ASV. However, in these cases, the *flaB* gene results did not exclude the presence of the species identified in by the V4 16S analysis in the analyzed samples. This also applied to the clade grouping of the previously unknown V4 16S variants (ASV10-12, ASV14). In this case, the *flaB* gene sequences indicated the presence of *B*. *garinii* and *B*. *lusitaniae*. In this regard, due to the presence of *B. burgdorferi* s.l. V4 16S sequence variants that could not be unambiguously assigned to the species, further analyses were based on sequencing data from *flaB* amplicons.

### 3.4. Presence of Babesia spp. DNA in Ixodes ricinus

The overall prevalence of *Babesia* spp. in host-seeking *I. ricinus* ticks was 4.4% (45/1029), and the infection was noticed in all developmental stages (Table 2), with the highest prevalence found in females and the lowest in nymphs (7.3% vs. 2.4%, respectively; χ^2^ test: *p* = 0.001). *Babesia*-infected ticks occurred in four out of five locations (range between 0.8% and 9.8%). Among three identified *Babesia* species, *Ba. canis* (2.6%) followed by *Ba. microti* (1.4%) were the most prevalent, and *Ba. venatorum* (0.4%) was the rarest species. These three species were noted in adult females and males, whereas *Ba. venatorum* was absent in nymphs.

In total, 6.0 % (76/1268) of feeding female *I. ricinus* ticks were positive for the presence of *Babesia* DNA (Table 2). The highest infection rate was noted in ticks collected from dogs (6.8%) and undefined animals (5.7%), and the lowest in cat-derived ticks (3.9%), but the difference was statistically insignificant. Three *Babesia* species were identified; two of them, *Ba. canis* and *Ba. microti*, reached similar infection rates (2.8% and 2.2%, respectively), whereas *Ba. venatorum* was the rarest species (1.0%). Ticks infected with these three species were confirmed in all animal groups. The infection levels of *Ba. canis* and *Ba. microti* in ticks derived both from dogs and undefined pets were similar (3.2%, 2.5% and 2.7%, 2.0%, respectively), with the lowest level for *Ba. venatorum* (1.0% and 0.7%, respectively). The latter prevailed (2.0%) in cat-derived ticks, followed by *Ba. microti* (1.3%) and *Ba. canis* (0.7%), which was found only in one female tick.

All obtained *Ba. canis* sequences demonstrated 100% similarity to *Ba. canis* isolates detected in the blood of infected dogs from Poland (e.g., GenBank: EU622793, KT844903), as well as in questing *I. ricinus* ticks (GenBank: MF797820). *Babesia microti* sequences were identical to the zoonotic *Ba. microti* “Jena/Germany” genotype (GenBank acc. no. EF413181), whereas *Ba. venatorum* sequences showed 100% homology to isolates found in patients in Italy and Austria (AY046575), and also in an asymptomatic patient in Poland (KP072001). 

### 3.5. Co-Occurrence of Borrelia burgdorferi s.l. and Babesia spp. DNA in Ixodes ricinus 

Overall, of the 2297 *I. ricinus* ticks, 10.7% (*n* = 246) tested positive for a single pathogen, and 1.0% (*n* = 24) yielded dual infections. Of the 149 ticks positive for *B. burgdorferi* s.l. DNA, mono-infections were identified in 83.8% (*n* = 125) and co-occurrence with *Babesia* spp. DNA in 16.1% ticks. Of the 121 ticks infected with *Babesia* species, 80.2% (*n* = 97) were single infections, and 19.8% were co-infected with *B. burgdorferi* s.l. Among host-seeking ticks, co-occurrence of *Borrelia* and *Babesia* was identified in 1.1% (11/1029) of adult ticks. None of the 460 nymphs were concurrently infected with both pathogens (Table 2). *Babesia canis* was the most prevalent species in co-infected ticks (72.7%; 8/11) and was confirmed in four (0.4%) co-infections with *B. afzelii* and in four (0.4%) with *B. garinii*. In the remaining three female ticks, *B. lusitaniae* co-occurred with *Ba. venatorum* (*n* = 2; 0.2%) and *Ba. microti* (*n* = 1; 0.1%).

Among feeding female ticks, 1.0% (13/1268) were co-infected with both pathogens. *Babesia microti* was the most prevalent species in all co-infections (92.3%; 12/13). Most co-infections of this species were recorded with *B. afzelii* (10/13). Furthermore, *Ba. microti* co-occurred with *B. spielmanii* and *B. garinii* in two other ticks, and *Ba. canis* co-occurred with *B. garinii* only in one female tick. 

### 3.6. Co-Occurrence of Borrelia burgdorferi s.l. and Borrelia miyamotoi in Ixodes ricinus 

Of the ninety host-seeking ticks positive for *B. burgdorferi* s.l., six (6.7%) were co-infected with *B. miyamotoi* spirochetes identified in our previous study [25]. *Borrelia afzelii* was the most common and occurred in three co-infections (two nymphs and one male), followed by *B. garinii* (two nymphs) and *B. lusitaniae* (one female). None of the 59 *B. burgdorferi* s.l.-positive feeding ticks yielded concurrent *B. miyamotoi.*

## 4. Discussion

In this study, we described the prevalence of *B. burgdorferi* s.l. and Babesia spp. found in mono- and double infections among I. ricinus ticks occurring in urban areas of the city of Poznań. We focused on two tick groups: (i) a group of host-seeking ticks collected from vegetation, and (ii) a group of feeding ticks removed from pet animals, dogs and cats.

According to a review by Hansford et al. [32], the mean *Borrelia* prevalence in questing ticks in urban green areas across Europe was 17.3% (range: 3.1% to 38.1%). In our study, 8.7% of ticks (range: 0.0% to 40.6%) yielded borreliae. Comparable or higher mean infection rates were found in urban *I. ricinus* populations of several European cities from neighboring countries. For example, in Slovakia, the prevalence of *B. burgdorferi* s.l. ranged from 6.8% to 15.3% in parks of Bratislava [33,34], and in agglomerations of Košice and Bardejov, it reached 10.2% [35]. In the Czech Republic, 12.1% of ticks collected in parks in Brno and 13.2% in Ostrava city yielded spirochetes [36,37]. Furthermore, in the city parks of Vilnius, Latvia, and in recreational areas of Hanover, Germany, 25% of ticks yielded *B. burgdorferi* s.l. [38,39]. Comprehensive studies on host-seeking ticks infected with *B. burgdorferi* s.l. in strictly urban agglomerations in Poland are relatively rare. In city forests and parks of Warsaw, the mean prevalence of *Borrelia* spirochaetes was 10.9% [6], and in the Tri-City agglomeration area of Gdańsk, Gdynia, and Sopot, it reached 12.4% [40]. To date, the highest levels of *Borrelia* infections have been described in green areas of the city Białystok (25.7%) and Olsztyn (27.4%) [41,42]. In our study, the highest prevalence of 40.3% (28/69) was recorded in ticks of the Citadel Park, the largest municipal park strictly in the center of the city. Although the number of tested ticks was limited, this high infection level may result from the lack of roe deer, which is considered to eliminate *B. burgdorferi* s.l. in feeding ticks [43]. 

The overall prevalence of *B. burgdorferi* s.l. found in ticks from pet animals (4.7%) was almost two-fold lower than in host-seeking ticks (8.7%). Comparable prevalences were described in the Netherlands [44,45] and Austria (4.8% and 5.2%, respectively) [46]. Higher infection rates in *I. ricinus*, mainly from dogs, were recorded in Latvia (10.7%), Germany (11.6%), Finland (11.8%), Norway (14%), and Denmark (15%) [47,48,49,50,51]. So far, the highest prevalences in *I. ricinus* from dogs have been found in two city agglomerations: Wrocław (21.7%) in the southwest [52] and Olsztyn (34.4%) in the northeast of Poland [53].

*Borrelia afzelii* and *B. garinii* proved to be the most prevalent species among infected ticks both from vegetation and pets. Our results are in agreement with the frequency of main *Borrelia* species infecting host-seeking *I. ricinus* ticks in central Europe [54] and with a report by Skotarczak [55] documenting that *B. afzelii* and *B. garinii* are the most common species in dogs. Dogs can develop infection of *B. burgdorferi* s.l. and exhibit the presence of antibodies, but unlike humans, they rarely get sick [55,56]. The distribution of Borrelia species in our study may be explained by species composition of wild hosts. The predominant *B. afzelii*, together with *B. spielmanii*, are maintained in enzootic cycles associated with small rodents and medium-sized mammals, including hedgehogs [57,58,59]. We found *B. spielmanii* only in three feeding females, which confirms the rarity and highly focal distribution of this spirochete. The mentioned groups of mammals were observed in our study locations, with a predominance of small rodents. In the present research, *B. afzelii* prevailed in feeding ticks (3.7%). This spirochete was also the most prevalent (3.6%), followed by *B. garinii* (1.7%), *B. valaisiana* (1.4%), and *B. spielmanii* (1.4%) in ticks from dogs in Latvia [50]. A reverse pattern, with a predominance of *B. garinii* over *B. afzelii* (28.1% vs. 3.2%), was observed in ticks from dogs in northeastern Poland [53]. Avian-associated *B. garinii* and *B. valaisiana* [60] were present in both infected tick groups, with almost a fourfold higher prevalence of *B. garinii* in ticks from vegetation in comparison to feeding ticks (42.2% vs. 11.2%). The lizard-associated *B. lusitaniae* [61] prevailed in host-seeking ticks compared to pet-derived ticks (10% vs. 1.7%). The finding of this spirochete only at the Rusałka lake suggests its highly focal distribution. 

In this study, the method of amplification and sequencing of the V4 region in 16S rRNA was not specific enough to clearly identify the obtained *B. burgdorferi* s.l. sequences with species as displayed on the phylogenetic tree. The 16S rRNA region had different sequences of V4 for some species identified by the flaB gene; therefore, a complex analysis including other marker genes is necessary to correctly assign 16S rDNA sequences to the species.

We detected *Babesia* DNA in 4.4% of host-seeking ticks, which were recorded in four out of five locations (range: 0.8% to 9.8%). The meta-analysis by Onyiche et al. [62] estimated the overall prevalence of babesiae in questing *I. ricinus* in Europe at 2.1%. Our results agree with infection rates found in the city Białystok (3.7%) [42] and within the Tri-City agglomeration (4.5%) [63]. Lower prevalences (range: 0.4% to 0.5%) were reported in ticks tested in Bavarian public parks [64] or in urban Bielański Forest (0.8%) in Warsaw [65]. 

*Babesia* DNA was identified in 6.0% of feeding ticks, with a higher prevalence in ticks from dogs (6.8%) compared to those from cats (3.9%). Stensvold et al. [49] documented a prevalence of 8.0% in dog-derived ticks in Denmark. Lower prevalences of *Babesia* spp. in pet-derived ticks (usually from dogs) were 0.8% in the Netherlands [45], 1.0% in Finland [51], 1.4% (62/4316) in the United Kingdom [66], 2.5% in Germany [48], and 4.7% in Latvia [50]. The highest infection rates in ticks feeding upon dogs (66.8%) and cats (15.4%) were reported in southern Poland [67].

In Europe, *I. ricinus* is involved in the transmission cycles of *Ba. divergens*, *Ba. venatorum*, *Ba. microti*, and *Ba. capreoli*, of which the first three are considered as human pathogens [8]. In our study, three species, including *Ba. microti*, *Ba. venatorum*, and *Ba. canis*, were identified in both groups of ticks. The finding of the first two species might explain the availability of ticks to suitable reservoir hosts of both parasites. In Europe, *Ba. microti* infects small rodents [68]. The reservoir host for *Ba. venatorum* is the roe deer, and this species was observed in at least four out of five study sites. However, we did not find *Ba. divergens*, the main etiological agent of human babesiosis, in European patients [21]. Since cattle are regarded as the reservoir hosts for *Ba. divergens*, their absence in urban areas of Poznań may explain why we failed to find the pathogen. In our study, *Ba. canis* and *Ba. microti* were the most prevalent species in feeding (2.6% and 1.4%, respectively) and host-seeking ticks (2.8% and 2.2%, respectively), whereas *Ba. venatorum* was the rarest piroplasm. Although the role of *I. ricinus* in the transmission of *Ba. canis* remains unclear, this parasite has already been found in questing ticks in northern Poland (range: 0.2% to 5.8%) [41,63,69], in Slovakia, and the Czech Republic (range: 0.2% to 1.4%) [70,71]. In our previous study, we found that 10.2% of ticks in forest ecosystems in west-central Poland were positive for *Ba. canis* DNA, the highest prevalence ever reported for this tick [22]. European *Ba. canis* strains belong to type A or type B, which induce different clinical symptoms of canine babesiosis [72]. In this previous and the current study, all *Ba. canis* obtained sequences represented the milder type A. In Europe, *Ba. canis* infections in *I. ricinus* from dogs were previously documented only in Latvia and Russia, with prevalences of 1.0% and 3.8%, respectively [50,73], and the prevalence of 3.2% in our study is comparable with those data. In Europe, most *Babesia* infections in *I. ricinus* collected from companion animals are caused by *Ba. microti* and *Ba. venatorum*. The overall prevalence of 2.2% of *Ba. microti* in feeding ticks in our study was lower compared to a 5.7% infection rate in dog-derived ticks in Warsaw, which yielded the *Ba. microti* Gray strain pathogenic to humans (GenBank: AY693840) [74]. In Europe, the highest prevalences of *Ba. microti* in this group of ticks were reported in southeastern (42.6%) and southern (24.3%) Poland [67,75]. In the case of *Ba. venatorum*, the highest prevalence of 1.6% was recorded in dog-derived ticks in Latvia [50]. According to Onyiche et al. [62], *Ba. microti* is considered to be the most common *Babesia* species in questing *I. ricinus* in eastern Europe. In our study, *Ba. microti* (1.4%) was the second most prevalent species following *Ba. canis*. Much higher infection rates of *Ba. microti* in ticks from urban areas were 2.6% in northern [40], 6.5% in central [76], and 26.4% in southern Poland [77]. *Babesia venatorum*, which infected only 0.4% of host-seeking ticks in our study, as well as in Bavarian public parks [64], also reached low infection rates of 0.8% in central [65] and 0.9% in northern Poland [69]. 

In Europe, more than 60 cases of human babesiosis have been caused mainly by *Ba. divergens*, with five cases attributed to *Ba. venatorum* and eleven autochthonous cases to *Ba. microti*, most of which were identified in Poland [21,78]. Two European genotypes of *Ba. microti* can infect humans, but they are less infectious or pathogenic to humans than those in the United States. The first *Ba. microti* genotype (strain) “Jena/Germany” (EF413181) is closely related to the USA genotypes (Clade 1) including, e.g., the Gray strain isolated from a patient in Massachusetts (AY693840), responsible for most cases of human babesiosis worldwide. The second *Ba. microti* genotype, the ‘Munich’ type (AB071177), is widely distributed in Europe and belongs to Clade 3 [79]. This genotype was originally thought to be non-zoonotic; however, it has recently been identified in six patients with mild and asymptomatic infections in Poland [80] and in one from Spain [81]. However, its pathological potential remains uncertain [78]. It should be noted that the *Ba microti* sequences identified in our study were identical to the *Ba. microti* genotype “Jena/Germany” that is considered pathogenic to humans [78]. In Poland, Welc-Falęciak et al. [82] found the same genotype in two asymptomatic patients. Furthermore, among 1.3% of *Babesia*-infected *I. ricinus* removed from humans in Poland, most showed a high similarity (>99%) to the *Ba. microti* strain Jena, followed by *Ba. venatorum* [83]. Of note is that the sequences of *Ba. venatorum* found in the two mentioned studies, as well as in ours, were identical to those isolated from splenectomized patients in Italy and Austria [84], and to an asymptomatic patient from Poland [85].

Altogether, out of 121 *Babesia*-positive ticks, 19.8% were simultaneously *Borrelia*-positive. We found almost identical prevalences of co-infections with *B. burgdorferi* s.l. and *Babesia* spp. in host-seeking (1.1%) as well as in feeding ticks (1.0%). To the best of our knowledge, this is the first report documenting the co-occurrence of both pathogens in ticks from companion animals in Europe. In Poland, co-infection rates of both pathogens in host-seeking ticks were described in the north (range: 0.3% to 0.6%) [40,86], in the east (1.6%) [87], and in the northeast (2.8%) [42]. In the present study, *Ba. canis* prevailed in co-infections mostly with *B. afzelii* and *B. garinii* in host-seeking ticks, whereas *Ba. microti* dominated predominantly with *B. afzelii* in ticks from pets. The prevalence of 0.9% (*n* = 9) *Ba. canis* and *B. burgdorferi* s.l. co-infections in host-seeking ticks in our study is consistent with a previous Polish study, in which 1.0% of 104 ticks showed a co-infection of *Ba. canis* with *B. afzelii* [88]. Recently, a fatal case of a dog co-infected with *Ba. canis* and *B. burgdorferi* s.l. was diagnosed in Romania [89]. Hildebrand et al. [90] found 1.6% of co-infections of *Ba. microti* followed by *Ba. divergens*, mostly with *B. afzelii* and *B. garinii* in questing ticks in Middle Germany, whereas *Ba. microti* and *B. afzelii* prevailed in co-infected ticks from humans [83].

Taking into account the mentioned studies, in host-seeking *I. ricinus*, *Ba. microti* seems to be the most prevalent species in co-infections with *B. afzelii* and *B. garinii*. Co-occurrence of these pathogens is of significant importance from a medical point of view. In humans, they may affect the clinical course of disease, especially in non-immunocompetent patients, and might be difficult to differentiate, since both infections induce often nonspecific symptoms, including fever, fatigue, and flu-like illness [91,92]. Jabłońska et al. [93] described a symptomatic case of babesiosis and LB in a Polish immunocompetent patient after travelling to Canada and the USA. Furthermore, in a study of 24 tick-exposed individuals from southeastern Poland, a piroplasm 98.9% homologous with *Ba. divergens* and *Ba. venatorum* was detected in one person concurrently seropositive for *B. burgdorferi* s.l. [94]. In another Polish report, Pańczuk et al. [95] also found a single *B. burgdorferi* s.l. and *Ba. microti* co-infection among foresters with significant levels of IgG anti-*B. burgdorferi* antibodies.

Using our previous results regarding the presence of *B. miyamotoi* spirochetes [25] in the same group of host-seeking ticks, we found that 0.6% of 1029 individuals yielded concurrently DNA of *B. burgdorferi* s.l., with predominance of *B. afzelii* and *B. garinii*. Co-occurrence of both pathogens was also documented in 0.4% and 1.4% of adult *I. ricinus* collected from vegetation in France [96] and eastern Poland [87], respectively. 

In conclusion, the finding of a predominance of two pathogenic species of spirochetes, *B. afzelii* and *B. garinii*, and the detection in both groups of ticks of the sequences *Ba. microti* and *Ba. venatorum*, which are identical to human pathogenic strains, indicate that people using urban green spaces are at risk of contracting LB and/or babesiosis. The occurrence of double infections, even with a low prevalence of 1.0%, is both clinically and epidemiologically significant and indicates that they pose a challenge for differential diagnosis in patients with acute febrile disease after contact with a tick. Furthermore, the finding of *Ba. canis* in both groups of *I. ricinus* suggests that this species could potentially be involved in the circulation of this piroplasm in areas of western central Poland, where the competent vector *Dermacentor reticulatus* is absent or rare. If this is the case, urban areas may pose a potential risk of dogs acquiring babesiosis.

## Figures and Tables

**Table 1:** The prevalence of *Borrelia burgdorferi* s.l. and *Babesia* spp. in single and double infections found in 1268 feeding *I. ricinus* female ticks removed from three groups of pet animals surveyed in veterinary clinics of the city of Poznań.

	Dogs (*n* = 609)	Cats (*n* = 117)	Undefined hosts (*n* = 389)	Total (%)
***Borrelia* spp.**				
*B. afzelii*	26 (3.7)	5 (3.3)	16 (4.0)	47 (3.7)
*B. garinii*	4 (0.6)	0	3 (0.7)	7 (0.6)
*B. spielmanii*	3 (0.4)	0	0	3 (0.2)
*B. lusitaniae*	1 (0.1)	0	0	1 (0.1)
*B. valaisiana*	0	0	1	1 (0.1)
Total	34/711 (4.8)	5/153 (3.3)	20/404 (5.0)	59/1268 (4.7)
***Babesia* spp.**				
*Babesia canis*	23 (3.2)	1 (0.7)	11 (2.7)	35 (2.8)
*Babesia microti*	18 (2.5)	2 (1.3)	8 (2.0)	28 (2.2)
*Babesia venatorum*	7 (1.0)	3 (2.0)	3 (0.7)	13 (1.0)
Total	48/711 (6.8)	6/153 (3.9)	22/404 (5.7)	76/1268 (6.0)
***co-infections***				
*B. afzelii + Ba. microti*	5	0	5	10 (0.8)
*B. spielmanii + Ba. microti*	1	0	0	1 (0.1)
*B. garinii + Ba. microti*	0	0	1	1 (0.1)
*B. garinii + Ba. canis*	0	1	0	1 (0.1)
Total	6	1	6	13/1268 (1.0)

## Data Availability

The data presented in this study are available on request from the corresponding author.

## Supplementary Materials

The following supporting information can be downloaded at: https://www.mdpi.com/article/10.3390/pathogens13040307/s1, Table S1. Sequences of the V4 16S rDNA region used in this study; Figure S1. Bayesian phylogenetic analysis of amplicon sequence variants (ASV) of the V4 region of the 16S rRNA gene found in this study. Numbers near branches show support values (PP); only values > 50% are present.
