# Supplementary material for: Co-Occurrence of Borrelia burgdorferi Sensu Lato and Babesia spp. DNA in Ixodes ricinus Ticks Collected from Vegetation and Pets in the City of Poznań, Poland
Source: Pathogens. 2024 Apr 10;13(4):307. doi: 10.3390/pathogens13040307 (PMC11054194; doi:10.3390/pathogens13040307)

Figure S1. Bayesian phylogenetic analysis of amplicon sequence variants (ASV) of the V4 region of the 16S rRNA gene found in this study. Numbers near branches show support values (PP); only values >50% are present.

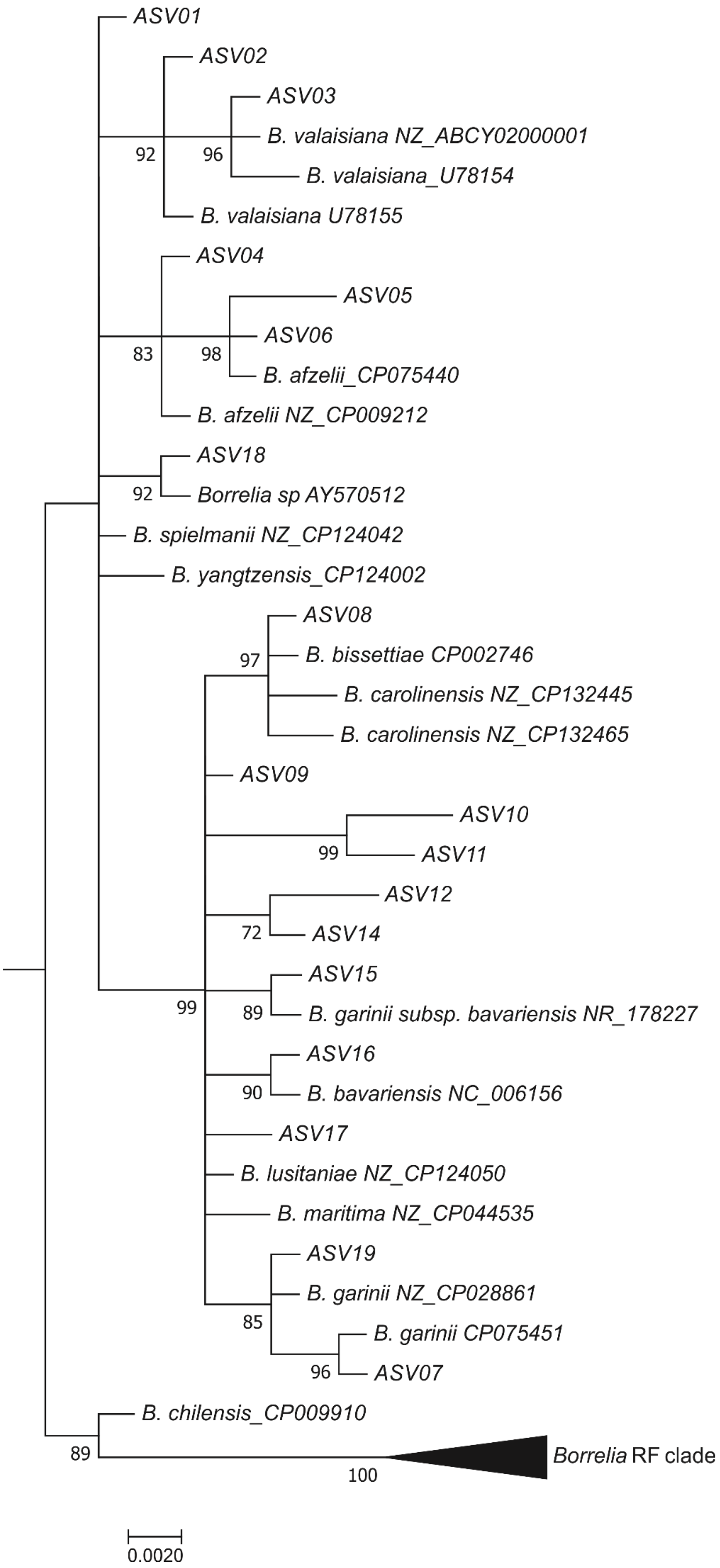

Supplement: Supplementary file 1 [file pathogens-13-00307-s001.zip › Figure S1.pdf]
