# Supplementary material for: Co-Occurrence of Borrelia burgdorferi Sensu Lato and Babesia spp. DNA in Ixodes ricinus Ticks Collected from Vegetation and Pets in the City of Poznań, Poland
Source: Pathogens. 2024 Apr 10;13(4):307. doi: 10.3390/pathogens13040307 (PMC11054194; doi:10.3390/pathogens13040307)
Supplement: Supplementary file 1 [file pathogens-13-00307-s001.zip › Table S1.pdf]

Table S1. Sequences of the V4 16S rDNA region used in this study.

**Species**	**GenBank acc. no.**
ASV01	PP406298
ASV02	PP406293
ASV03	PP406303
ASV04	PP406292
ASV05	PP406300
ASV06	PP406302
ASV07	PP406304
ASV08	PP406305
ASV09	PP406296
ASV10	PP406297
ASV11	PP406299
ASV12	PP406294
ASV14	PP406295
ASV15	PP406306
ASV16	PP406290
ASV17	PP406291
ASV18	PP407998
ASV19	PP407999
B. afzelii	CP075440
B. afzelii	NZ_CP009212
B. anserina	CP013704
B. bavariensis	NC_6156
B. bissettiae	CP002746
B. carolinensis	NZ_CP132445
B. carolinensis	NZ_CP132465
B. chilensis	CP009910
B. coriaceae	NZ_CP005745
B. crocidurae	NZ_CP004267
B. duttonii	NZ_AZIT01000001
B. garinii	CP075451
B. garinii	NZ_CP028861
B. garinii subsp. *bavariensis*	NR_178227
B. hermsii	NZ_CP011060
B. lusitaniae	NZ_CP124050
B. maritima	NZ_CP044535
B. miyamotoi	MZ918969
B. miyamotoi	MZ918974
B. miyamotoi	MZ918983
B. miyamotoi	MZ918981
B. parkeri	NZ_CP007022
B. recurrentis	NC_11244
B. spielmanii	NZ_CP124042

B. turcica	NZ_CP028884
B. turicatae	NC_8710
B. valaisiana	NZ_ABCY02000001
B. valaisiana	U78154
B. valaisiana	U78155
B. yangtzensis	CP124002
Borrelia sp.	AY570512
